# Insights into trypanosomiasis transmission: Age, infection rates, and bloodmeal analysis of *Glossina fuscipes fuscipes* in N.W. Uganda

**DOI:** 10.1371/journal.pntd.0011805

**Published:** 2024-10-31

**Authors:** Lucas J. Cunningham, Johan Esterhuizen, John W. Hargrove, Mike Lehane, Jennifer Lord, Jessica Lingley, T. N. Clement Mangwiro, Mercy Opiyo, Iñaki Tirados, Steve J. Torr

**Affiliations:** 1 Department of Vector Biology, Liverpool School of Tropical Medicine, Liverpool, United Kingdom; 2 Agricultural Research Council—Onderstepoort Veterinary Research, Pretoria, South Africa; 3 DSI-NRF Centre for Epidemiological Modelling and Analysis, University of Stellenbosch, Stellenbosch, South Africa; 4 Bindura University of Science Education, Bindura, Zimbabwe; 5 Malaria Elimination Initiative, Institute of Global Health Sciences, University of California San Francisco, San Francisco, California, United States of America; 6 Centro de Investigação em Saúde de Manhiça (CISM), Manhiça, Mozambique; University of Cincinnati, UNITED STATES OF AMERICA

## Abstract

**Background:**

Tsetse flies (*Glossina*) transmit species of *Trypanosoma* which cause human African trypanosomiasis (HAT) and animal African trypanosomiasis (AAT). Understanding the epidemiology of this disease and controlling the vector rationally requires analysis of the abundance, age structure, infection rates and feeding patterns of tsetse populations.

**Methods:**

We analysed a population of *G*. *fuscipes fuscipes* in the Koboko district of Uganda. Seasonal variation in the abundance of tsetse was assessed from the numbers of tsetse caught in pyramidal traps. The age structure of the population was assessed by dissecting female tsetse to estimate their ovarian categories. Classical and PCR-based methods were utilised to determine the presence of the three major pathogenic species of salivarian trypanosomes: *T*. *vivax*, *T*. *congolense* and *T*. *brucei* in a subset (n = 2369) of flies. Further, bloodmeal analysis was carried out using PCR to amplify and sequence a portion of the vertebrate cytb gene.

**Results:**

The abundance and age structure of tsetse populations were relatively stable and a slight seasonal four-fold variation in abundance appeared to be correlated with rainfall. Analyses of age structure suggests a low natural daily mortality of 1.75% (1.62–1.88). Infection rates estimated were significantly greater (1.9–9.3 times) using the PCR-based method compared to the classical dissection-based method. Positive rates for *T*. *brucei* sl, *T*. *congolense* and *T*. *vivax* were 1.6% (1.32–2.24), 2.4% (1.83–3.11and 2.0% (1.46–2.63), respectively by PCR. The majority of bloodmeals were identified as cattle (39%, 30.5–47.8) and human (37%, 28.4–45.6).

**Conclusion:**

The seasonally stable abundance, low mortality rate and high proportion of bloodmeals from humans may explain, in part, why this district has historically been a focus of sleeping sickness. Additionally, the high rates of cattle feeding indicate insecticide treated cattle may prove to be a useful vector control strategy in the area.

## Author summary

Tsetse flies (*Glossina*) transmit African trypanosomiasis in humans (‘sleeping sickness’) and livestock (‘nagana’). Quantifying the abundance, age structure, infection rates and host preferences of tsetse populations is important for designing and monitoring vector control operations. Tsetse were caught along the Kochi river in Koboko in N.W. Uganda. Age of tsetse was determined through ovarian ageing while dissection and PCR were both used for xenomonitoring. Feeding behaviour was inferred through bloodmeal analysis via sequence analysis.

Analyses of these results showed that natural mortality was low (1.75%/day) among adult females and seasonal variation in abundance and age structure was relatively slight. PCR proved significantly more sensitive as a xenomonitoring tool than the classical dissection method with positive rates of 1.6%, 2.4% and 2% for *T*. *brucei* sl, *T*. *congolense* and *T*. *vivax*, respectively. The most important hosts were cattle followed by humans which provided 39% and 37% of bloodmeals respectively.

The stable population in combination with a high rate of human blood feeding may help explain why this area has been a historic focus of sleeping sickness. The high proportion of bloodmeals from cattle suggest that insecticide treated cattle may prove to be an effective vector control strategy.

## Background

Tsetse flies (*Glossina*) transmit species of *Trypanosoma* which cause human African trypanosomiasis (HAT) and animal African trypanosomiasis (AAT). Across Central and West Africa, riverine tsetse (e.g., *G*. *palpalis*, *G*. *fuscipes*) transmit *T*. *brucei gambiense* which causes Gambian HAT (gHAT), a chronic and anthroponotic form of the disease. In East and Southern Africa, savanna tsetse (e.g., *G*. *morsitans*, *G*. *pallidipes*) transmit *T*. *b*. *rhodesiense* which causes Rhodesian HAT (rHAT), an acute zoonosis. Both forms of HAT are fatal unless treated with drugs. Between 2000 and 2021, the annual number of cases of gHAT and rHAT reported globally by the World Health Organisation has declined by 98% (747/25841) and 86% (55/709) respectively [1].

Uganda is the only country with both forms of HAT and has experienced severe epidemics of both over the last century. The most recent epidemic occurred towards the end of the last century and numbers have declined markedly since then. During 1990–1999, for instance, the mean number of cases of gHAT and rHAT reported annually in Uganda were 1384 and 516 cases/year, respectively, compared to 5 and 25 cases/year, respectively, for the period 2012–2021 [1]. The decline in cases of gHAT has been achieved through a combination of mass screening and treatment of the human population [2], coupled with use of Tiny Targets to control tsetse. Control of rHAT cases has been achieved through mass treatment of cattle with trypanocides and insecticides [3, 4]; in Uganda, cattle are important reservoir hosts for *T*. *b*. *rhodesiense* [5] and a source of bloodmeals for tsetse [6]. Recognising the progress made against gHAT, the WHO provided formal validation in 2022 that gHAT has been eliminated as a public health problem in Uganda [7].

For the first trial of Tiny Targets in northwest Uganda, the impact of the intervention was assessed by comparing catches from monitoring traps deployed in areas with or without targets [8]. The traps in the non-intervention area were deployed along the river Kochi in Koboko district. While the main purpose of these traps was to provide a measure of tsetse abundance in the absence of Tiny Targets, the catches also provide an opportunity to quantify seasonal variation in the abundance and composition of a tsetse population. Assessing both provides insights into the dynamics of a tsetse population and the underlying causes of changes in abundance. Variation in age structure is also important for quantitative studies of infection rates and transmission dynamics [9–12]. Flies can be aged using either the wing fray method or more accurately using ovarian dissection [13,14].

In addition to monitoring the distribution and abundance of tsetse populations to assess the impact of an intervention [8, 15–18], tsetse are sometimes analysed for the presence of pathogenic subspecies of *T*. *brucei* [17]. As gHAT becomes a relatively rare disease, monitoring tsetse for evidence of transmission by the presence of *T*. *b*. *gambiense* may become an important part of disease surveillance [19].

Classically, detection and identification of trypanosomes was based on microscopic examination of tsetse [20] but this approach faces three important limitations. First, a fly observed to have trypanosomes present in the salivary glands and midgut is presumed to be infected with *T*. *brucei*. However, this method is unable to distinguish between *T*. *brucei brucei*, which is not pathogenic to humans, and the pathogenic subspecies *T*. *b*. *gambiense* and *T*. *b*. *rhodesiense*. Second, the presence of trypanosomes in the midgut only may signify an immature infection with *T*. *brucei* or *T*. *congolense*. Third, mature co-infections of, say, *T*. *congolense* and *T*. *brucei* would be incorrectly identified as being an infection with *T*. *brucei* only. These limitations have been highlighted in empirical studies of savanna tsetse in Tanzania [21] and riverine tsetse in Côte d’Ivoire [22]. Following comparison of dissection and PCR-based methods, Lehane *et al*. [21] concluded that “Given that so much of our understanding of the epidemiology of trypanosomes in tsetse flies is based on the Lloyd & Johnson (1924) dissection technique these results are potentially alarming and the study needs to be repeated as a matter of urgency.”

Several studies of different species of tsetse, including for example, *G*. *longipalis* in Burkina Faso [23], *G*. *palpalis palpalis*, *G*. *p*. *gambiensis* and *G*. *tachinoides* in Côte d’Ivoire [22, 24–26], *G*. *p*. *gambiensis* in Guinea [27] and *G*. *pallidipes* in Zimbabwe [28] used PCR to characterise infections identified initially by dissection. Other studies have applied PCR-based methods to samples of tsetse that were not pre-screened by microscopic examination. Examples of this approach include studies of *G*. *p*. *palpalis* in Nigeria [29], *G*. *pallidipes* and *G*. *swynnertoni* in Tanzania [30], *G*. *f*. *quanzensis* in DRC [31] *G*. *f*. *fuscipes* in Uganda [32]. To date there has been no study that has screened both dissection positive and negative flies at the resolution of individual tissues. Such a high resolution approach would provide greater insights into the infection status of flies as well as better validate the sensitivity and specificity of PCR- and microscopy-based methods.

Another problem is that a mature, salivary gland, infection of *T*. *brucei* takes at least 15 days to develop after a fly feeds on an infected host [33]. This, coupled with the fact that traps tend to be biased towards the capture of older flies [34] means that the infection rate among trap-caught flies will tend to be greater than in the population as a whole.

Recognition of the limitations of dissection-based methods has led to greater use of PCR as a diagnostic tool [30, 32, 35]. However, these studies applied PCR-based methods to whole flies and hence were unable to distinguish tsetse with mature infections from immature ones, and it is only the former that are infectious. Moreover, detection of trypanosome DNA does not necessarily indicate the presence of an active infection in tsetse [36].

In the present study, we analysed catches from the monitoring traps along the river Kochi to address three research objectives. First, over a 15 month period we quantified monthly variation in the abundance, age structure and trypanosome prevalence of a natural population of *G*. *f*. *fuscipes* in northwest Uganda. Second, we compared dissection- and PCR-based estimates of trypanosome infections in a tsetse population sampled over a 15 month period in northern Uganda. Third, extraction of DNA from large numbers of tsetse to identify DNA from trypanosomes also provided material for us to identify important hosts in the diet of the local tsetse population. Taken together, the various sorts of data we produced are useful in the interpretation of sampling operations and the planning of control in N-W Uganda, and are of practical and theoretical interest elsewhere.

## Methods

### Study site

All field studies were conducted, between April 2013 and July 2014 along the Kochi River in the Koboko district of north-west Uganda [15]. The area is predominantly agricultural with crops such as cassava, tobacco, millet and sesame being farmed. Potential hosts of tsetse include various livestock species (cattle, goats, sheep and pigs) and monitor lizards [37]. Koboko and the surrounding districts have long been a focus of sleeping sickness, with reports dating back to 1905 [38]. The primary vector of *T*. *b*. *gambiense* in this district is *G*. *f*. *fuscipes* [8], a riverine species of tsetse.

### Tsetse sampling

Pyramidal traps [39] were placed individually at four sites (3.45–3.47N, 31.03–31.09E) along the Kochi river, <1m from the river’s edge and >100 m from each other (S1 Fig). These traps formed part of a network of traps deployed initially to monitor the impact of Tiny Targets on tsetse populations. The traps along the Kochi River monitored tsetse numbers in an area where Tiny Targets were not deployed and hence provide data on a natural, uncontrolled, population of tsetse. Traps were checked twice daily, at ~07:30 h and ~15:30 h. Tsetse were brought back to the laboratory within 2h of collection in a cool box to reduce mortality from desiccation and heat stress. In the laboratory, the samples were dissected on the day of collection or the following morning.

### Tsetse dissection

Male and female tsetse were dissected to determine the presence of trypanosomes. Female flies were also subjected to ovarian dissection to determine their age. The dissections were carried out by trained technicians using fine forceps and Zeiss Stemi 2000 dissection microscopes. Salivary glands, midgut and mouthparts were dissected out and then examined at 200× and 400× magnification using a compound microscope fitted with a dark-field filter. Tissues (e.g., salivary glands, midgut, mouthparts) were identified as positive if live, i.e., mobile, trypanosomes were observed within them. Infections were assigned putatively to a species of *Trypanosoma* based on the tissue(s) where trypanosomes were found.

Following the classical methodology [20], the putative identity of infections was based on observation of trypanosomes in the mouthparts, midgut and salivary glands, Where trypanosomes were observed in the midgut and salivary glands, the fly was deemed to be infected with *T*. *brucei* sensu lato. Presence of trypanosomes in the midgut and mouthparts indicated an infection with *T*. *congolense*, and trypanosomes in the mouthparts only were categorised being infected with *T*. *vivax*. An infection of the midgut only could be an immature infection of *T*. *brucei* s.l. or *T*. *congolense*.

### *Molecular identification of* Trypanosoma

Following dissection and scoring for infection and age, the mouthparts, salivary glands and midgut were each stored individually in 60μl of absolute ethanol, for subsequent molecular analysis; tissues from flies were stored in this manner regardless of whether trypanosomes had been found by microscopic examination. The samples were stored in 96-well PCR plates, with each plate holding tissues from 32 flies. Of the 6869 tsetse dissected, a subsample of 2370 was selected for screening using species-specific multiplex ITS PCR [40], using PCR product size to determine trypanosomes species identity. A further subsample of 768 flies was selected for analysis aimed at identifying the source(s) of their bloodmeals. Due to the use of pyramidal traps, which attract hungry flies, the majority of flies caught lacked a visible bloodmeal, therefore flies were screened for bloodmeals blind.

### DNA Extraction

DNA was extracted from the samples as follows. The plate was spun at ~6,000 rpm for 30 seconds and then the lids from the plates were removed and the ethanol evaporated off in an oven at 56°C for 1–2 hours. After evaporation, 135μl of DNA extraction buffer was added. The buffer comprised 1xTE/5% Chelex/ 1% proteinase K (20mg/ml). To elute the DNA, samples were incubated at 56°C for an hour, followed by a second incubation step at 93°C for 30min. The supernatant (100μl) was then pipetted off into a new 96-well plate, being careful not to pick up any of the Chelex beads.

### PCR protocols

*Trypanosoma*. For each fly, 2μl of DNA eluate was taken from the three tissues and pooled together, effectively reconstituting the fly. This pooled sample was then screened with the multiplex ITS primers [40], to screen for *T*. *congolense*, *T*. *brucei* and *T*. *vivax*. Any positive fly then had eluates from the three tissues tested separately using the same primers. In the present study, we do not report analyses to identify the subspecies of *T*. *brucei*. Our previous work suggests that all infections with *T*. *brucei* s.l. were *T*. *b*. *brucei* [40].

*Blood meal analysis*. The DNA eluate from a subsample comprising 768 midguts was screened using sequencing primers, designed to amplify vertebrate DNA by targeting a ~470bp region of the cytochrome b gene. Due to the type of trap used, only hungry, un-fed, flies were caught. Therefore, it was not possible to select “fed-flies” to undergo bloodmeal analysis but rather flies were randomly selected from both wet and dry seasons and residual DNA from previous feeds was amplified in 17% of flies tested. This allowed the host DNA in the bloodmeal to be targeted and amplified and sequenced [41]. The primer sequences are as follows; forward ‘TACCATGAGGACAAATATCATTCTG’, reverse ‘CCTCCTAGTTTGTTAGGGATTG ATCG’. These primers amplified a ~470bp region of the vertebrate mitochondrial genome. Once amplified the PCR product was then cleaned using the QIAquick PCR purification kit (Qiagen, Manchester, UK) and sequenced by SourceBioScience (Nottingham, UK). Sequences were cleaned and aligned using MEGA software [42] resulting in a 410bp clean sequence. Following on from this the sequences were screened against the NCBI database of vertebrate genomes using the against basic local alignment tool (BLAST) [43]. Species ID was based on achieving ≥97% percentage identity with reference sequences.

### Environmental data

To assist in the interpretation of temporal variation in the catches, age structure and infection rates of tsetse, we collected local estimates of monthly rainfall and mean temperature. These were obtained from the Global Precipitation Climatology Centre (GPCC) and HCN_CAMS Gridded 2m Temperature (Land) data, respectively. All data were provided by NOAA PSL, Boulder, Colorado, USA, from their website at https://psl.noaa.gov.

### Data analysis

All analyses were performed using the open-source statistical software programme R [44].

*Tsetse Catches*. Catches of tsetse per trap per day varied between months. We hypothesised that this was due in part to seasonal patterns in temperature and/or rainfall. Using the ‘glmmADMB’ package, we fitted a generalised linear mixed effects model (glmm) to the daily catches of male and female tsetse. We specified a negative binomial distribution for catches, with site and day of collection as random effects and month as a fixed effect. Using the model outputs, we estimated the mean daily catches and their 95% confidence intervals for each month. To assess the impact of temperature and total rainfall, we also produced glmms of catch with monthly rainfall and/or temperature as explanatory variables. The significance of these variables was assessed by analyses of deviance (ANODEV) using the ‘Anova’ function. In addition to assessing relationships of monthly catch, and the simultaneous mean temperature and total rainfall, we also assessed whether rainfall and temperature from earlier months had an effect; previous studies have found relationships between abundance of tsetse and the mean saturation deficit, likely affected by rainfall, from earlier months [45].

*Age structure*. Previous studies of the age structure of tsetse have shown large and statistically significant inter-monthly variations in the distribution of Ovarian Categories (OC) [46]. To discover whether similar variation occurred with *G*. *f*. *fuscipes* in Uganda, we assessed the statistical difference in the monthly distributions of OCs for all consecutive months by Chi-squared tests. We also calculated the mean OC for each month and used Tukey’s multiple comparison test to assess the statistical significance of differences between means. To elucidate trends over a series of months, we carried out regression analyses to assess changes in the mean Ovarian Category and time (months) as a continuous variable.

*Adult mortality rates*. We used a maximum likelihood method to estimate adult female mortality from the distribution of ovarian ages among samples of females captured in traps [47]. The derivation of the method is provided here in S1 Text.

*Infection rates*. To assess the statistical significance of differences in the percentage of tsetse observed to be (i) infected with *Trypanosoma* as estimated by dissection and (ii) positive by PCR by logistical regression, we fitted data using a general linear model (glm) with a binomial error distribution and a logit link function, using method as a factor. The significance of explanatory factors was assessed by analyses of deviance (ANODEV) using the ‘Anova’ function.

For all analyses of catches, age structure and mortality rates, the 95% error limits are shown.

## Results

### Catches of tsetse

**Fig 1:**
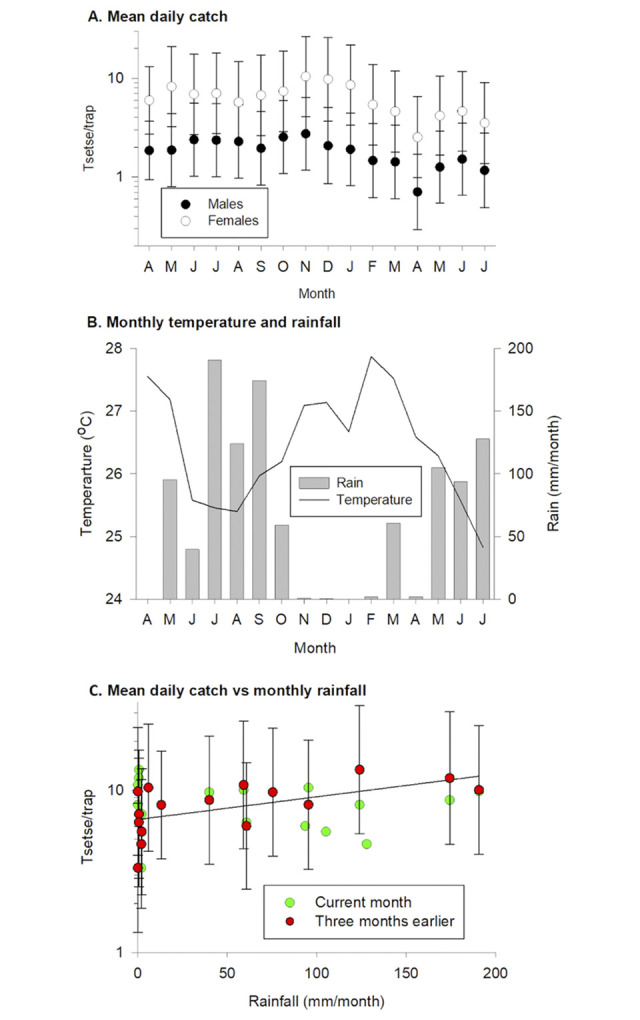
(A) Mean daily catch of male and female tsetse, (B) monthly mean temperature and total rainfall between April 2013 and July 2014 and (C) scatterplot of mean daily catch of tsetse (males and females combined) against monthly rainfall. For C, catches for each month are plotted against the rainfall for that month or that three months earlier. Line shows catch predicted from fitting glmm to rainfall three months earlier.

Between April 2013 and July 2014, the four traps caught a total of 12 512 tsetse across 238 trapping days. The mean daily catches of male and female tsetse peaked in November 2013 (Fig 1A) and the lowest numbers were caught in April 2014; mean daily catches of males and females showed similar peaks and troughs, but the mean catch and range were greater for females (2.5–10.4) than males (0.7–2.7). Fitting a glmm to the data showed that Month (P<0.001, Deviance = 678.08., df = 1) and Sex (P<0.001, Deviance = 69.96.14, df = 15)] were statistically significant factors but there was no statistically significant interaction between them (P = 0.515, Deviance = 14.14, df = 15). Catches of males and females were pooled for analyses of the relationships between abundance, monthly rainfall and mean monthly temperature. The results showed that there was no significant correlation between mean catch and the current temperature (P = 0.282, Deviance = 1.16, df = 1) or rainfall (P = 0.689, Deviance = 0.16, df = 1) but for rainfall only there was a significant relationship between catch and the total rainfall from earlier months, with the strongest correlation being between catch and the rainfall from three months earlier (P<0.01, Deviance = 20.6, df = 1). Analysis of the pooled data on sex showed that the predicted mean daily catch increased significantly with the rainfall from three months earlier (P<0.01, Deviance = 19.72, df = 1). Catches increased from 6.6 (3.40–12.84) tsetse/trap/day for dry (0 mm rain/month) months to 12.2 (5.96–24.98) tsetse/trap/day for the wettest (190.7 mm/month).

### Population age structure

**Fig 2:**
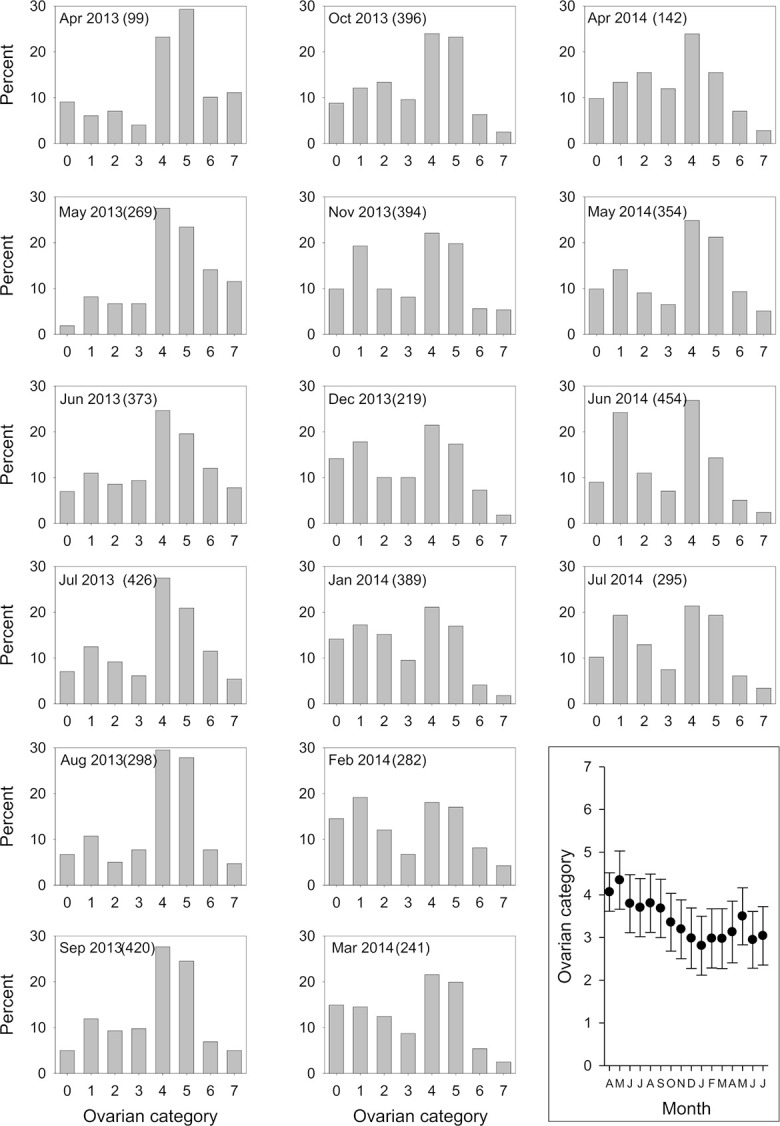
Percentage of tsetse in ovarian categories 0–7 for each month between April 2013 and July 2014. Inset shows mean ovarian category for each month. Numbers in brackets show the sample size for each month.

A total of 5051 female tsetse were dissected to determine their Ovarian Category (Fig 2). Comparing the distributions for all possible consecutive months showed that for most (12/15) contrasts there was no significant difference (Table 1). Two of the three significant contrasts occurred in the comparisons of May-June in 2013 and 2014 and the third was between October-November 2013. These changes were due largely to decreases in the percentage of tsetse from Ovarian Categories 5–7 and an increase in Ovarian Categories 0–3. The May-June changes in distribution were associated with a significant (Table 1) decrease in the mean Ovarian Category (Fig 2 inset). The May-June period marks the transition between the hot-dry and wet seasons (Fig 2B).

While the changes in age structure between consecutive months during the period June 2013-January 2014 were not statistically significant, there was a general and significant (P<0.001, Deviance = 321.8, df = 1) decline in mean OC (Slope = -0.146, SE = 0.0156) during this period. Conversely, for the period January-May, where the OC distributions of consecutive months were also not significantly different, there was a statistically significant trend over the period (P<0.001, Deviance = 81.1, df = 1) with mean OC increasing (slope = 0.155, SE = 0.0343) between January-May 2014. Overall, mean OC declined during the wet season and increased during the dry, but the inter-monthly variation was generally small.

**Table 1:** Statistical significance of differences in the distribution (χ^2^ test) and mean (Tukey test) of Ovarian Categories for consecutive months.

			χ^2^ test			Tukey comparisons of means		
Year	Month1	Month2	χ ^2^	df	P	Difference	95% CI	P
2013	Apr	May	13.7	7	0.058	0.24	-0.537–1.019	1.000
	May	Jun	16.3	7	**0.023**	-0.55	-1.075–0.017	**0.035**
	Jun	Jul	5.8	7	0.566	-0.09	-0.564–0.375	1.000
	Jul	Aug	11.7	7	0.112	0.10	-0.396–0.603	1.000
	Aug	Sep	7.3	7	0.401	-0.11	-0.614–0.389	1.000
	Sep	Oct	12.0	7	0.101	-0.34	-0.803–0.124	0.464
	Oct	Nov	14.8	7	**0.039**	-0.14	-0.609–0.333	1.000
	Nov	Dec	8.4	7	0.301	-0.24	-0.801–0.314	0.983
	Dec	Jan	5.6	7	0.592	-0.15	-0.706–0.411	1.000
2014	Jan	Feb	11.8	7	0.107	0.17	-0.346–0.689	0.999
	Feb	Mar	6.3	7	0.500	0.02	-0.564–0.596	1.000
	Mar	Apr	5.0	7	0.656	0.14	-0.557–0.842	1.000
	Apr	May	11.2	7	0.131	0.33	-0.326–0.988	0.940
	May	Jun	26.1	7	**<0.001**	-0.55	-1.017–0.078	**0.006**
	Jun	Jul	8.6	7	0.279	0.16	-0.335–0.655	0.999

Probabilities <0.05 are emboldened.

### Estimate of adult mortality

A global estimate of adult mortality, for the period June 2013—May 2014, was calculated using the maximum likelihood (ML) method described in S1 Text. The numbers of flies in ovarian categories 0 to 7+4n were 383, 564, 416. 334, 949, 815, 304 and 169, respectively, providing a mean mortality estimate for the 12-month period of 1.75% (1.63–1.88).

### Trypanosomes infection rates

A total of 1221 male and 5062 female tsetse were dissected and screened for trypanosomes using traditional microscopy methods. For males, trypanosomes were observed in 1.06% (13/1221, 0.57–1.81) of midguts, 0.08% (1/1221, 0.00–0.46) of salivary glands and 0.57% (7/1221, 0.23–1.18) of mouthparts. For females, the percentages were 1.50% (76/5062, 1.19–1.88), 0.12% (6/5062, 0.04–0.26) and 1.46% (74/5062, 1.15–1.83), respectively. One male (1/1221 = 0.08%, 0.002–0.455) and six females (6/5062 = 0.12%, 0.044–0.258) had trypanosomes in the midgut and salivary glands and hence were putative mature infections of *T*. *brucei s*.*l*.. One male (0.08%, 0.002–0.453) and 14 (0.28%, 0.151–1.464) female tsetse were putative mature infections of *T*. *congolense*, and six males (0.49%, 0.180–1.061) and 60 (1.19%, 0.907–1.525) females were classed as infections with *T*. *vivax*.

A sub-sample of 2369 tsetse (438 males, 1931 females) examined by microscopy between September 2013 and February 2014, were also screened using the mITS primers to identify tsetse positive for *T*. *brucei* s.l., *T*. *congolense* and *T*. *vivax* DNA. The results (Table 2) show that, for the midgut, salivary glands and mouthparts, the PCR-based method detected the presence of trypanosomes at a higher rate than microscopy. Over all combinations of sex and tissue, the numbers of positive tsetse detected by PCR were 1.8–9.3 times greater with PCR and the increases were statistically significant for all combinations apart from male mouthparts.

**Table 2:** Percentages of male (n = 438) or female (n = 1931) tsetse with trypanosomes detected by microscopy or PCR in the midgut (MG), salivary glands (SG) or mouthparts (MP), and the probabilities (P) that the percentages estimated by microscopy and PCR are not different (ANODEV).

			Microscopy		PCR				
Tissue	Sex	n	mean	95% CI	mean	95% CI	F	df	P
MG	male	438	**0.68**	0.141–1.989	**3.65**	2.102–5.865	10.0	1	0.002
	female	1931	**1.40**	0.923–2.028	**3.83**	3.021–4.787	23.2	1	<0.001
SG	male	438	**0.23**	0.006–1.265	**1.83**	0.792–3.567	6.3	1	0.012
	female	1931	**0.16**	0.032–0.453	**1.45**	0.966–2.089	23.4	1	<0.001
MP	male	438	**0.46**	0.055–1.640	**1.14**	0.372–2.644	1.3	1	0.247
	female	1931	**1.55**	1.051–2.210	**2.85**	2.153–3.691	7.6	1	0.006

The three female tsetse that had infected salivary glands detected by microscopy, and hence nominally infected with *T*. *brucei*, were in ovarian categories 4–6; none of the salivary glands of 890 females in ovarian categories 0–3 were observed by microscopy to be infected. On the other hand, PCR detected trypanosome DNA in 2 females from each of categories 0–2 and 3 females in category 3 (S1 Table). However, only two of the tsetse in ovarian category 3 were identified as being from *T*. *brucei;* the other seven specimens were identified as being *T*. *vivax* or *T*. *congolense*.

Comparing the identity of infections based on the presence or absence of trypanosomes in the mouthparts, midgut and salivary glands and PCR showed (Table 3) that of the 23 tsetse identified as being *T*. *vivax* by microscopy, 17 were positive by PCR and all of these were indeed infected with *T*. *vivax*, albeit four were mixed infections with either *T*. *brucei* or *T*. *congolense*. Six tsetse putatively identified, by microscopy, as being infected with *T*. *congolense* were PCR-positive for *T*. *congolense* (n = 4) or mixed infections with *T*. *brucei* (n = 2). Finally, the three tsetse nominally infected with *T*. *brucei* were identified by PCR as two mixed positives (*T*. *brucei* and *T*. *congolense*) and one *T*. *congolense* positive. Tsetse observed with infections in the midgut only (n = 20) were expected to have immature infections of *T*. *congolense* and/or *T*. *brucei*. Of these 20, 12 were successfully identified by PCR; 11 had one or both these species of *Trypanosoma*; and one was identified as *T*. *vivax*. In general therefore, the microscope- and PCR-based methods of identifying species of trypanosome infections were in broad agreement, but the PCR-based method was able to identify mixed infections.

**Table 3:** PCR-based identity of tsetse (n) putatively identified by microscopy.

Species	MP	MG	SG	PCR-based identity							
				n	None	Tv	Tc	Tb	Tv+Tb	Tv+Tc	Tb+Tc
*T*. *vivax* (Tv)				23	6	13	0	0	2	2	0
*T*. *congolense* (Tc)				6	0	0	4	0	0	0	2
*T*. *brucei* (Tb)				3	0	0	1	0	0	0	2
Tb and/or Tc				20	8	1	5	3	0	0	3

Classically, the putative identity of infections is based on observation of trypanosomes in the mouthparts (MP), midgut (MG) and salivary glands (SG) as indicated by the black shading in the table.

PCR-based analyses of infection can detect single and mixed infections of *T*. *vivax* (Tv), *T*. *congolense* and *T*. *brucei*. None indicates tsetse observed to be infected by microscopy but not by PCR.

For trypanosomes detected by PCR, the majority of positives were located where expected: *T*. *vivax* and/or *T*. *congolense* were detected in 83% (50/60) of PCR-positive mouthparts, *T*. *congolense* and/or *T*. *brucei* were detected in 68% (61/90) of PCR-positive midguts and *T*. *brucei* was detected in 14/36 PCR-positive salivary glands. However, we found many examples of species of *Trypanosoma* in unexpected places. For instance, DNA from *T*. *vivax* was detected in the midguts of 34 tsetse and *T*. *congolense* was detected in the salivary glands of 18 tsetse. These aberrant locations may be due to contamination during the dissection and processing of specimens. Evidence for this is provided by the observation that, of the 18 *T*. *congolense* positive salivary glands, only three occurred as a single tissue positive. For the other 15, DNA from *T*. *congolense* was detected in the mouthparts and/or midgut, where *T*. *congolense* is expected, as well as the salivary glands. In other cases, the detection of DNA from *Trypanosoma* species in unexpected tissues may be a consequence of ‘natural’ contamination of trypanosome DNA within the fly. For instance, it is not inconceivable that *T*. *vivax* in the mouthparts may become dislodged and ingested into the midgut. The 34 tsetse with midguts that were positive for *T*. *vivax* also had mouthparts that were positive for *T*. *vivax*.

Ignoring the locations of trypanosomes within the flies, the infection rates estimated by PCR-based analyses were 1.64% (1.321–2.244) for *T*. *brucei*, 2.41% (1.827–3.106) for *T*. *congolense* and 1.98% (1.461–2.630) for *T*. *vivax*. However, if we regard true infections as being only those where DNA from each species was detected in the correct organs, then the mature infection rates were 0.17% (0.046–0.432) for *T*. *brucei* s.l., 0.46% (0.232–0.829) for *T*. *congolense* and 1.48% (1.031–2.049) for *T*. *vivax*. The percentages for immature infections of *T*. *brucei* s.l. and *T*. *congolense* were 0.80% (0.484–1.250) and 1.48% (1.031–2.049).

### Blood meals

**Fig 3:**
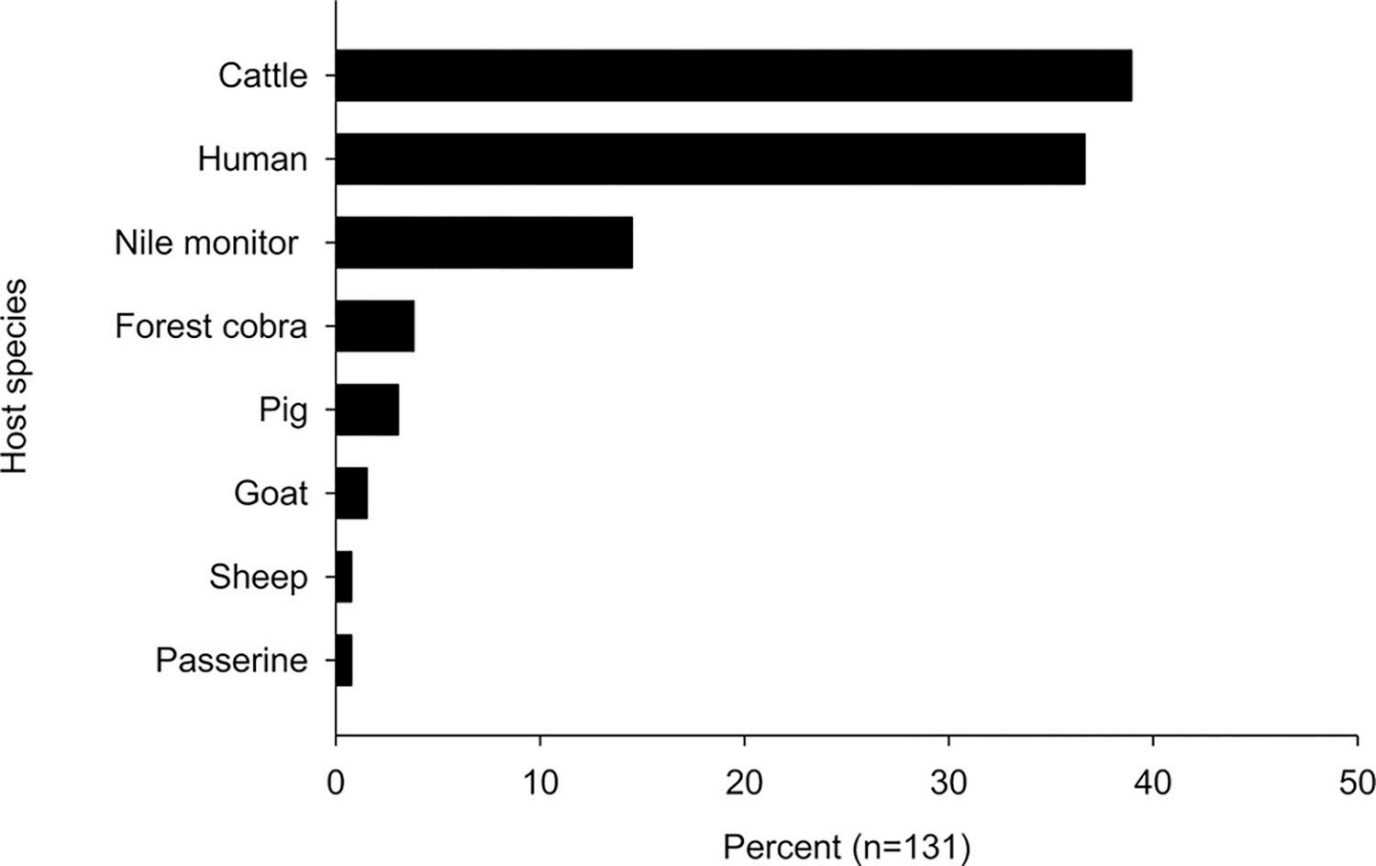
Percent of tsetse bloodmeals from human and animal host species.

A subsample of 768 tsetse was analysed for the source of their bloodmeals; of these 131 bloodmeals (17.1%, 14.46–19.91) were identified successfully, with all samples that provided good quality sequence reads achieving percentage identity to reference samples of 99.21%-100%, with the exception of *Varanus niloticus*, which scored 92% sequence identity, despite good quality sequence reads. The sequences obtained from the bloodmeals for the different hosts have been submitted to the NCBI database with accession numbers for these submissions given in S2 Table. The results (Fig 3) showed that cattle were the most common host (39%, 30.5–47.8) closely followed by humans (37%, 28.4–45.6). We believe that this is the first recording of Forest cobra (*Naja melanoleuca*) being identified as a host of tsetse (4%, 1.3–8.7).

## Discussion

### Population dynamics

For *G*. *fuscipes* in north-west Uganda, the abundance of tsetse ranged between a maximum mean daily catch of 13.4 tsetse/trap in November 2013 and a minimum of 3.3 tsetse/trap in April 2014. There was no statistically significant variation in the ratio of males:females. The relatively modest four-fold seasonal variation in abundance is similar to that observed for *G*. *f*. *fuscipes* in Uganda [48, 49], Ethiopia [50], and Kenya [8]. Recent analyses of the population genetics of *G*. *f*. *fuscipes* in Uganda also suggest that the populations are relatively stable[51].

The apparent stability of *G*. *f*. *fuscipes* contrasts with the much greater fluctuations in the abundance of savanna tsetse. In Zimbabwe for instance, catches of male *G*. *m*. *morsitans* and *G*. *pallidipes* in mopane woodland showed 35- and 13-fold differences between seasons respectively [52]. For females, the ratios were 2.5 and 7.5 respectively. Hence for these savanna species there is a large seasonal variation in numbers and also the ratio of males:females. Studies of other populations of savanna tsetse in East Africa also show large (>10-fold) variations in abundance [53,54].

While the fluctuations in catches were relatively slight, the change appeared to be correlated with rainfall 2–3 months earlier. Other studies have also found that rainfall [55], temperature [56] or saturation deficit and NDVI from earlier, rather than contemporaneous, months were correlated with the abundance or mortality rates of tsetse populations. This relationship demonstrates a lag in the rainfall and subsequent tsetse numbers two to three months later. This suggest an indirect relationship between the two which may be based on the effects of humidity on successful larval development in both the mother and in the soil. This is supported by early research showing that the rate of transpiration during the middle period of pupal development is directly related to the humidity that early larval stages were exposed to [57].

In keeping with the relatively stable numbers of tsetse, but contrasting with results for savanna tsetse [46], we found only slight seasonal variation in age structure. The only marked inter-month fluctuation seems to be associated with the onset of the rains at the end of the wet season (May-June). A potential explanation for the observed increase in the proportion of young tsetse at the beginning of the rains may be because pupae and/or teneral tsetse are susceptible to the dry conditions during the hot season (Dec-April). The survival of the pupae of *G*. *fuscipes* is reduced under dry conditions in comparison to savanna species of tsetse [58]. Poor survival of pupae and teneral tsetse may lead to an increase in the proportion of older tsetse in the population. The change in age structure might be greater than indicated by trap catches, since traps tend to catch only old flies [34].

Changes in pupal survival and humidity may explain the relationship between abundance of tsetse and rainfall 2–3 months earlier. There is a lag between the onset or cessation of the rains and changes in relative humidity [55]. As more humid conditions become established, there is increased survival of pupae leading to an increase in the numbers of adults about a month later, i.e. the duration of the pupal period. Catches of tsetse from traps are biased towards older flies; the most numerous age class for most months was Ovarian Category 4 (Fig 2) where flies would be >40 days old.

Oher potential causes of variation in catches may relate to seasonal changes in the area over which tsetse disperse [49] and the impact of flooding on the pupal population. During the dry season, tsetse may concentrate closer to the river and hence catches from traps might increase. We observed a decrease in catches as the dry season progressed. Flooding might be expected to decrease the proportion of young tsetse during the wet season whereas we observed the opposite.

The female adult mortality rate of 1.75% per day, estimated using the ML method applied to samples of flies collected over a 12-month period, is markedly lower than estimates averaging about 2.4% per day, resulting from the analysis of female *G*. *pallidipes* from Zimbabwe [46]. This is to be expected, given the harsher climatic conditions in the Zambezi Valley. Analysis of the Zimbabwe data showed that application of the method to samples of flies collected over monthly periods produced estimates that made little sense, given meteorological conditions at the times of capture. For example, mortality was found to take a minimum in December, one of the hottest months of the year; this is contrary to mark-recapture estimates indicating that mortality rates increase significantly with increasing temperature. This problem has been ascribed, primarily, to the fact that the estimation of mortality from age distribution involves the assumption of an underlying stable age distribution. As pointed out previously [59], it seems unlikely, given the large oscillations in temperature, in the Zambezi Valley, that these distribution are ever stable. Given the more equable climate of Uganda it seemed possible that there might be scope for estimating mortality over periods of time of the order of one month. In practice, however, it was found that the mortality estimates, as with the Zimbabwe situation, showed no sensible correlation with meteorological conditions.

### Infection rates

By comparing estimates of infection prevalence estimated by microscopic examination of tsetse, and by a PCR-based method, we tested the null hypothesis that, for *G*. *f*. *fuscipes*, the two methods provide identical estimates of *Trypanosoma* infection prevalence. We reject this hypothesis since our results show that the latter was significantly more sensitive, particularly for infections of the midgut and salivary glands. Over all combinations of sex and tissues, the number of nominally infected flies were 1.8–9.3 times greater with PCR. Our results accord with studies of other species of savanna and riverine tsetse in, for example, Tanzania [21] and Cote d’Ivoire [22] respectively, but extend the findings to another species of tsetse for all the major pathogenic species of *Trypanosoma*. Using the PCR-based method and ignoring the location where DNA was detected, infection rates for *T*. *brucei*, *T*. *congolense* and *T*. *vivax* were 1.64%, 2.41% and 1.98%, respectively. However if we regard epidemiologically significant infections as being those where DNA is detected in the correct organs then the respective rates were 0.17%, 0.46% and 1.48%. The infection rates estimated using microscopic examination of the midgut, mouthparts and salivary glands were 0.11%, 0.24% and 1.05%. Hence, while PCR detects trypanosome DNA in a higher percentage of tsetse, the infection rates relevant for, say, epidemiological modelling are not grossly dissimilar. If the aim of a study is to detect the presence of *Trypanosoma* species circulating in a population of tsetse, then PCR is more sensitive, particularly for *T*. *brucei* s.l. and *T*. *congolense*.

The presence of DNA from a *Trypanosoma* species in the correct organ(s) does not necessarily mean that the fly is infected; DNA from trypanosomes that have been killed by immune responses of the fly can be detected for a period after their ingestion [36]. Moreover, the presence of *T*. *brucei* or *T*. *congolense* in the midgut does not indicate an infectious tsetse.

The low rate of infection observed in our study accords with previous findings, showing a low prevalence of salivary gland positives, equating to ~1/1000 flies having a mature *T*. *brucei* sl infection [20, 28]. Previous studies of infection rates of tsetse populations in the Adjumani and Moyo districts of northwest Uganda conducted in reported infection rates (n = 272) of 0.74%, 2.6% and 6.6% for *T*. *brucei* s.l., *T*.*congolense* and *T*. *vivax* respectively [32]. A more recent study conducted in 2019 in Arua and Maracha districts reported that 3.6% (13/360) of tsetse dissected were observed to be infected with trypanosomes, with 3.1% (11/360) having mouthpart infections, suggestive of *T*. *vivax*, and 0.6% (2/360) had midgut infections and hence potentially being immature infections with *T*. *congolense* and/or *T*. *brucei* s.l. [60]. These infection rates are in broad agreement with ours.

### Bloodmeals

Our results show that cattle and humans were the most important sources of bloodmeals. The range of hosts is similar to results from earlier studies [6,61] but the proportion of meals from cattle and humans is higher than previously reported for *G*. *fuscipes* from this area of Uganda. We are not aware of estimates of the absolute or relative densities of livestock in 2013–2014 for Koboko district, but estimates for 2006 suggest that densities decline in the order goats (27,000), cattle (10,000), sheep (7,000) and pigs (200) [62]. The national census of 2014 estimated that the human population in Koboko district was 208,000 [63]. On the basis of these numbers, it is unsurprising that humans are an important part of the diet of the tsetse population. The importance of cattle in the diet may reflect their large size and relative unresponsiveness to biting tsetse in comparison to sheep and goats [64].

The high proportion of meals from humans combined with the low mortality rate is important because these factors contribute to higher rates of transmission. Building on Roger’s models of trypanosomiasis [65], many HAT models assume that natural tsetse mortality rates are 3%/day and the proportion of meals from humans is 30%. Substituting values of 1.75% and 37%, respectively, in Roger’s model increases R_0_ from 2.7, 388.2 and 64.4 for *T*. *brucei*, *T*. *congolense* and *T*. *vivax* (Table 3 in [65]) to 3.06, 610.8 and 114.9, respectively.

The high proportion of meals from cattle suggests that regular treatment of cattle with pyrethroids would be an effective method of controlling tsetse in this area.

### Limitations

*Estimates of infection rates*. We detected trypanosome DNA in tissues where they are not expected: *T*. *vivax* was detected in the midgut of 34 tsetse and *T*. *congolense* in the salivary glands. At least some of these aberrant locations appear to be due to contamination by the dissectors despite our efforts to avoid this. The contamination was between tissues of the same fly rather than between flies and our procedures were less effective at preventing the former. While there is evidence of some contamination affecting our estimates, there is also evidence that detection of trypanosome DNA in unexpected tissues is also a natural phenomenon. For instance, a relatively high proportion of midguts with *T*. *vivax* were associated with the detection of *T*. *vivax* in the mouthparts. The simplest explanation of this is the passage of *T*. *vivax* parasites, or DNA, from an infected proboscis into the midgut during feeding and the subsequent detection of this transient DNA by PCR.

*Bloodmeal analysis*. The study utilised only a single gene target for bloodmeal identification, which was successful in identifying the majority of vertebrate-cytb positive flies down to the species level, with a cut-off of 97%. The exception being *V*. *niloticus*, which only had a 92% identity with reference sequences on the NCBI database at the species level. An additional gene target site for bloodmeal identification would likely have helped resolve this issue, as there is only a single submission for *V*. *niloticus* on the NCBI database that covers the same cytb region that we have targeted, meaning the available reference sequences likely do not represent the full genomic diversity if *V*. *niloticus*, which may help to explain the low identity score, despite, high-quality sequence reads being generated. This is further complicated by the fact that *V*. *niloticus* is a species complex and therefore the molecular diversity is likely to be high between the different sub-groups [66]. A final limitation with regards to the bloodmeal is the potential that the demographics of available hosts may have changed in the intervening years, as such, the proportion of bloodmeals taken from different hosts may have changed since the study was carried out. However, it is likely that the predominant hosts (humans and cattle), still remain in similar proportions in the area, as there has not been any major event that would contribute to a decrease in human numbers of drastic change in culture, i.e. civil unrest, disease or severe ecological disaster.

*Seasonal variations in abundance and age structure*. The study was conducted over 15 months in a relatively small geographical area; we use the results to infer large-scale seasonal patterns in populations of *G*. *f*. *fuscipes*, and the underlying causes of these changes. We are not aware of previous studies of this or longer duration which analysed abundance, age structure and diet. Nonetheless, we recognise that stronger inferences could be produced by conducting the studies over several years and over a wider geographical area. Data being produced by vector control operations conducted in north-west Uganda [15] will produce longer series over larger areas and future analyses of these data will allow us to build on the present results.

*Estimates of mortality rates*. The age composition of female tsetse shows some unexpected features, most noticeably the very large proportions of flies in ovarian category 4+4*n* and 5+4*n*.These are composite categories, which include flies that have ovulated 4, 8, 12, … etc and 5, 9, 13 …. etc times, and it is thus unsurprising that they contain greater proportions than found in category 3. However, since we expect numbers to decline monotonically with age, the differences between the proportions of flies in category 4+4*n*, or 5+4*n*, and those in category 3, should be less than the proportion in category 7+4*n*. In fact, however, the reverse is true for every one of the 16 months of the study–as is obvious from Fig 2. These results are consistent with suggestions that there is a marked sampling bias against young tsetse (<Ovarian Category 4), as has been reported for savanna tsetse [34]. Such biases undermine confidence in our approach to estimating mortality rates. Research on the causes and consequence of biases in sampling methods are required.

## Conclusion

Our study has shown that PCR-based methods provide a more sensitive way of detecting and quantifying infection of tsetse with *T*. *congolense*, *T*. *brucei* s.l. and *T*. *vivax*.

During the 15-month period of our study, there were only slight changes in the abundance, age structure and infection rates of tsetse. The changes that were observed were correlated with rainfall in previous months, which we suggest affected the survival of pupae.

The high proportion of meals from cattle suggest that insecticide-treated cattle should make a significant contribution to the control of HAT and AAT.

## Supporting information

S1 Text Maximum likelihood estimation of adult tsetse mortality from ovarian age data. (DOCX)

S1 Fig Locations of the four sampling sites where pyramidal traps were deployed along the Kochi river in the Koboko district of N.W. Uganda. Figure generated using GIS with base layer of rivers derived from: Stanton, M.C *et al* 2018 [67].
(TIF)

S2 Fig Copy of Excel file used to estimate mortality among adult female tsetse from the observed distribution of ovarian categories among flies captured in the field. For the data in this example we estimate a mortality rate of 1.75% per day (95% confidence interval 1.63% - 1.88%).
(TIF)

S1 Table Number of female tsetse with trypanosomes detected by microscopy or PCR in the midgut, salivary glands or mouthparts. (DOCX)

S2 Table Accession numbers for host bloodmeals detected, genotypes for each host varied across different hosts: cattle (*n* = 3), human (*n* = 7), forest cobra (*n* = 2), Nile monitor (*n* = 1), pig (*n* = 1), goat (*n* = 1) and sheep (*n* = 1). (DOCX)

S1 Data Raw tsetse population data used to generate results in this study. (XLSX)
