# Supplementary material for: Insights into trypanosomiasis transmission: Age, infection rates, and bloodmeal analysis of Glossina fuscipes fuscipes in N.W. Uganda
Source: PLoS Negl Trop Dis. 2024 Oct 31;18(10):e0011805. doi: 10.1371/journal.pntd.0011805 (PMC11556741; doi:10.1371/journal.pntd.0011805)
Supplement: S1 Text — (DOCX) [file pntd.0011805.s001.docx]

**Supporting Text:** Maximum likelihood estimation of adult tsetse mortality from ovarian age data.

We consider the case where it is not possible to identify the exact ovarian age for flies that have ovulated more than three times, so that one is working with pooled age categories for all of these older flies. We assume that there is no age-dependent sampling bias – except in the case of flies in ovarian category zero, which are under-represented and have always been excluded from such analyses in the past [1]. This method was first suggested by Hargrove [2] and has been used by Hargrove & Ackley [3] and Hargrove & Van Sickle [4], who also calculated 95% confidence intervals for the mortality estimates. The full derivation of the method is provided below since it is not available in any other publication.

For female tsetse, where it is not possible to distinguish between flies in categories 4, 8, 12, 16 ….. , 4+4*i i* = 0 - ∝, and similar statements hold for ovarian categories 5, 6 and 7. For flies which have ovulated *i*≥ 1 times, the probability (*p_i_*) that a randomly chosen fly is in ovarian category *i* is:

*p_i_*_­ =_ *φ^i^*^-1^(1- *φ*) *i* > 0 (1)

where *φ* is the probability that a fly survives a single pregnancy. This probability is assumed independent of the number of pregnancies already survived. To get the probability of a fly being in ovarian category 4+4*n* we must calculate the sum (*p*_4+4_*_n_*) of the probabilities for flies which have ovulated 4, 8 *etc*. times.

*p*_4_ = *φ*^3^(1- *φ*)

*p*_8_ = *φ*^7^(1- *φ*)

*p*_12_ = *φ*^11^(1- *φ*) (2)

and so on. The total probability is thus:

*p*_4+4_*_n_* = (1- *φ*)(*φ*^3^ + *φ*^7^ + *φ*^11^ + ….)

= (1- *φ*)(*φ*^3^(1 + *φ*^4^ + *φ*^8^ + ….)

= ((1- *φ*)*φ*^3^)/ (1- *φ*^4^) (3)

Similar calculations can be carried out for *p*_5+4_*_n_*, *p*_6+4_*_n_* and *p*_7+4_*_n_* and, generally:

*p_i_*_+4_*_n_* = ((1- *φ*)*φ*^i-1^)/(1- *φ*^4^) for 4 ≤ *i* ≤ 7 (4)

The likelihood, for a sample containing *n_i_* flies in each of the *i* categories, is now given by:

 (5)

For the maximum likelihood solution we must therefore solve

*x*_1_ (1+ *φ* + *φ*^2^ + *φ*^3^) – *x*_2_ (1- *φ*^4^) – 4*x*_3_ *φ*^4^ = 0 (6)

where (7)

This polynomial is solved for using Newton’s method, or other numerical analysis tools available in standard mathematical/statistical packages. Notice that an identical estimate of the mortality can be calculated by maximising the log likelihood (log *L*) numerically.

*Maximum likelihood estimation of the standard error of the mortality estimate*

The following development, carried out by John Van Sickle, was used to calculate 95% confidence interval for the maximum likelihood estimates of mortality derived from ovarian dissection data, as described above. These error estimate were first used by Hargrove & Van Sickle [4].

For the maximum likelihood estimation of a single parameter, ϕ, the standard error (SE) is given by:

SE(ϕ) = 1/ √(*I* (ϕ)) (8)

The function *I* (ϕ) is often called the Fisher information, defined as

*I* (ϕ) = - E[∂^2^*LL*/∂ϕ^2^] (9)

where: *LL* = log likelihood of the model

∂^2^(•)/∂ϕ^2^ = second derivative with respect to ϕ

E[•] = Expectation operator

We apply this approach to the Equation (1) of the mortality model, where the left-hand side gives the negative first derivative of the log-likelihood:

∂*LL*/∂ϕ = -*x*_1_(1 + ϕ + ϕ^2^ + ϕ^3^) + *x*_2_(1 - ϕ^4^) + 4 *x*_3_ϕ^4^ (10)

where *x*_1_, *x*_2_ and *x*_3_ are given by Equation (7).

The second derivative is then:

∂^2^*LL*/∂ϕ^2^ = -*x*_1_(1 + 2ϕ + 3ϕ^2^) - *x*_2_ 4ϕ^3^ + 16 *x*_3_ϕ^3^ (11)

Next, we take the expected value of the second derivative, which applies only to the values of *n_i_*, *i* = 1, 2, 3, 4+4*n*, … 7+4*n*, which are viewed as random variables, conditional on

Σ *n_i_* = *N* = total sample size

Thus, *I* (ϕ) = -E[∂^2^*LL*/∂ϕ^2^] = E[*x*_1_](1 + 2ϕ + 3ϕ^2^) + E[*x*_2_]4ϕ^3^ – 16 E[*x*_3_]ϕ^3^ (12)

where: $E[x_{1}] =\sum_{i=1}^{7} n_{i}$ = E[*N*] = *N* (13) $E[x_{2}] =\sum_{i=1}^{7} i(E[n_{i}])=(\sum_{i=1}^{7} ip_{i})N$ (14)

$E[x_{3}] =\sum_{i=4}^{7} (E[n_{i}])=(\sum_{i=4}^{7} p_{i})N$ (15)

In Equations 13 - 15, the values of *p_i_* are given by evaluating Equation (1) for ovarian ages 1, 2 and 3, and Equation (4) for ages 4+4*n* to 7+4*n*, using the estimate of ϕ, obtained from solving ∂*LL*/∂ϕ = 0, into those seven equations. We think of E[*n_i_*] = *p_i_N* as the number of flies predicted by the model to be in age class *i*, assuming the total count, *N*, is correct.

Finally, insert the values from Equations (13), (14) and (15) into Equation 12, again using the estimate of ϕ, to calculate ϕ, ϕ^2^ and ϕ^3^. This gives a numerical value for *I* (ϕ), and hence for the standard error of ϕ using Equation (1).

***Example data set***

We use, as the example, data comprising the ovarian ages of all female *G. f. fuscipes* captured during this study, in odour-baited traps deployed in northern Uganda, between June 2013 and May 2014. Using the ovarian dissection data, we use the Solver function in Excel to calculate ϕ by solving Equation (6) above with the values of *x*_1_, *x*_2_ and *x*_3_ calculated using the formulae given in Equation (7). Similarly, we use the age distribution, together with calculated value of ϕ to estimate the standard error of ϕ and, thereby, a 95% confidence interval for ϕ. These results are used to calculate the corresponding values of the daily mortality rate and its standard error and 95% confidence interval.

The steps involved are illustrated in (Figure S1), which is a copy of the Excel sheet where the calculations were carried out. The Excel sheet should be read in conjunction with the above derivations of the formulae for the estimated mortality and its error bounds.

**References**

1. Rogers DJ, Randolph SE, Kuzoe FAS. Local Variation in the Population-Dynamics of Glossina-Palpalis-Palpalis (Robineau-Desvoidy) (Diptera, Glossinidae) .1. Natural-Population Regulation. Bulletin of Entomological Research. 1984;74(3):403-23. PubMed PMID: WOS:A1984TK86500005.

2. Hargrove JW. Management of Insect Pests: Nuclear and Related Molecular and Genetic Techniques. Vienna: International Atomic Energy Agency; 1993.

3. Hargrove JW, Ackley SF. Mortality Estimates from Ovarian Age Distributions of the Tsetse Fly *Glossina pallidipes* Austen Sampled in Zimbabwe Suggest the Need for New Analytical Approaches. Bulletin of Entomological Research. 2015;105(3):294-304. doi: 10.1017/s0007485315000073. PubMed PMID: WOS:000354104000004.

4. Hargrove JW, Van Sickle J. Improved Models for the Relationship Between Age and the Probability of Trypanosome Infection in Female Tsetse, *Glossina pallidipes* Austen. Medical and Veterinary Entomology [*In press*]. 2023.
