# Supplementary figures and images for: Insights into trypanosomiasis transmission: Age, infection rates, and bloodmeal analysis of Glossina fuscipes fuscipes in N.W. Uganda
Source: PLoS Negl Trop Dis. 2024 Oct 31;18(10):e0011805. doi: 10.1371/journal.pntd.0011805 (PMC11556741; doi:10.1371/journal.pntd.0011805)

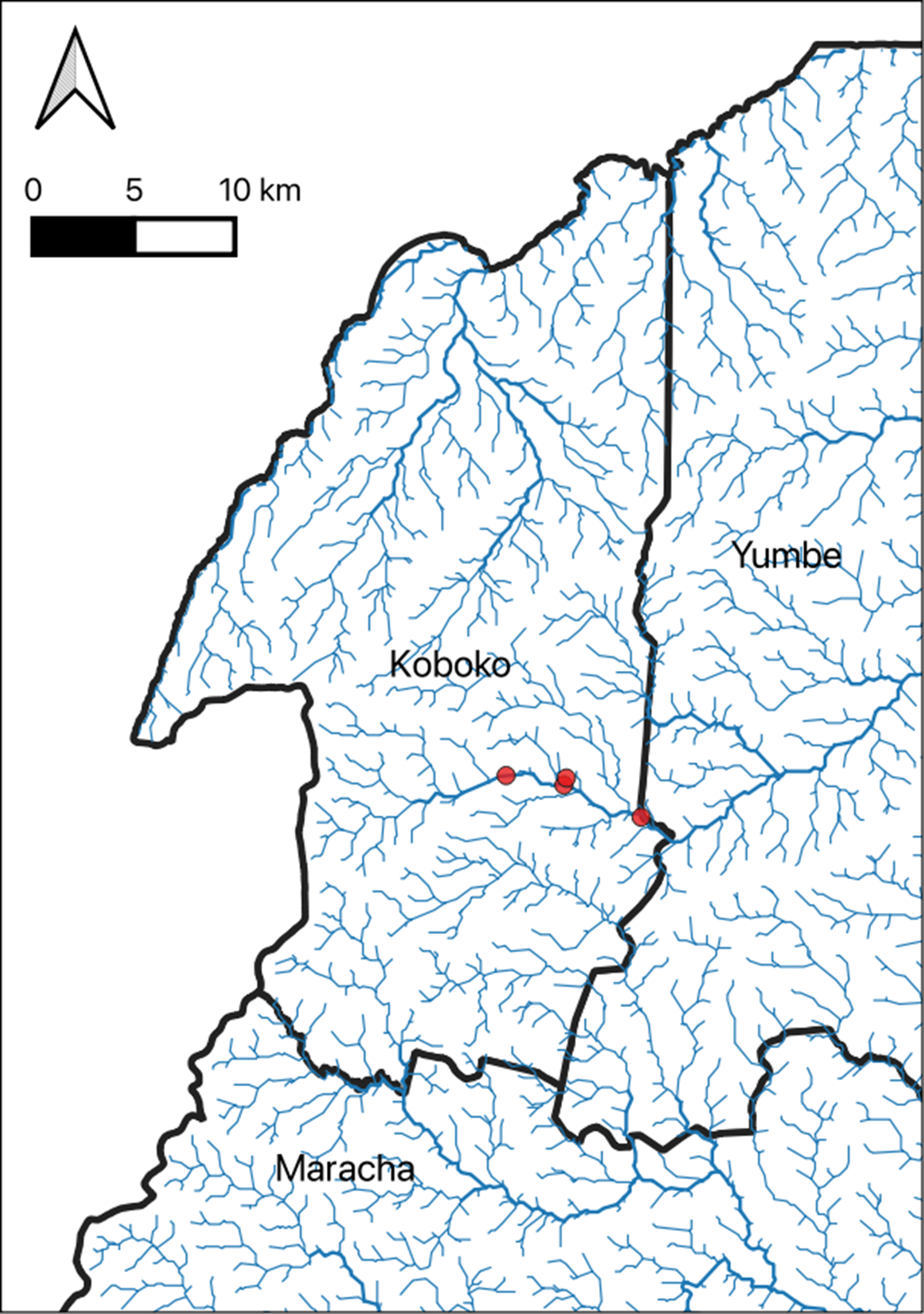

Supplement: S1 Fig — Figure generated using GIS with base layer of rivers derived from: Stanton, M.C et al 2018 [67]. (TIF) [file pntd.0011805.s002.tif]

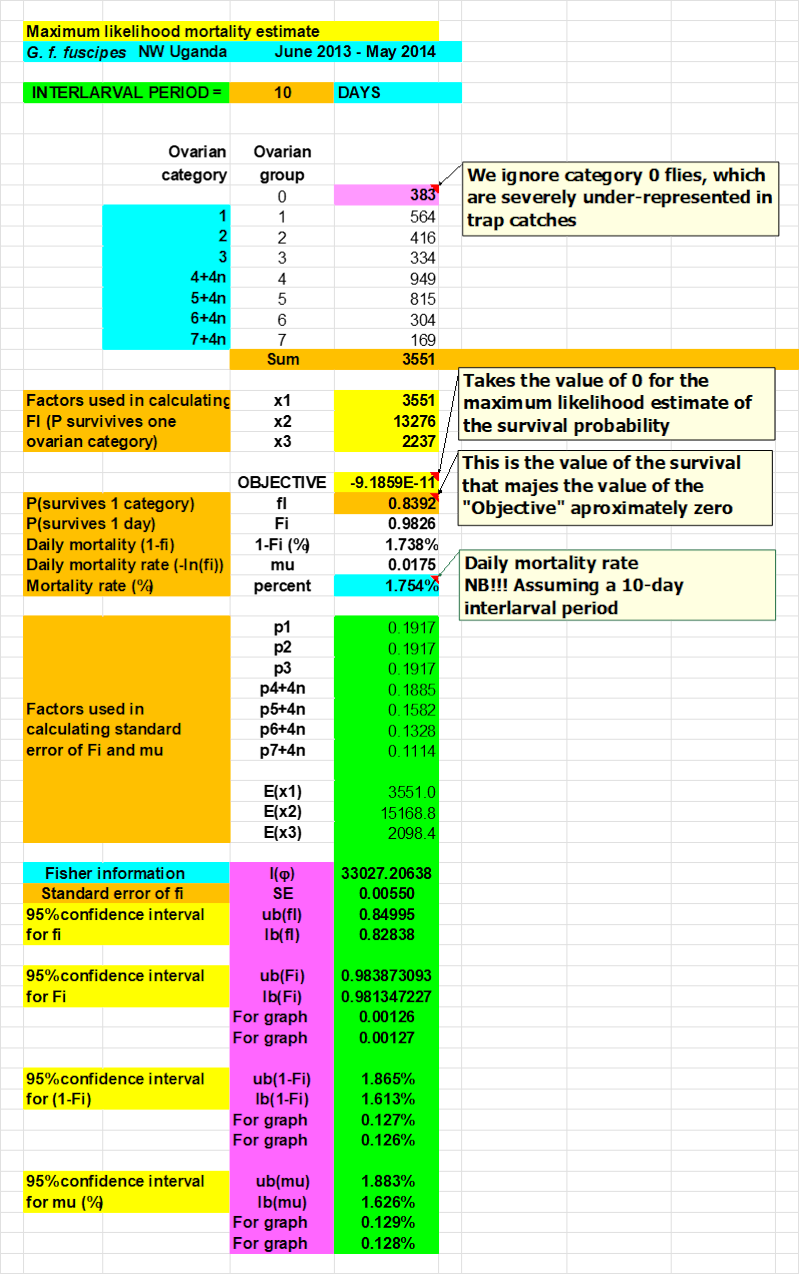

Supplement: S2 Fig — For the data in this example we estimate a mortality rate of 1.75% per day (95% confidence interval 1.63% - 1.88%). (TIF) [file pntd.0011805.s003.tif]
