# Supplementary material for: Insights into trypanosomiasis transmission: Age, infection rates, and bloodmeal analysis of Glossina fuscipes fuscipes in N.W. Uganda
Source: PLoS Negl Trop Dis. 2024 Oct 31;18(10):e0011805. doi: 10.1371/journal.pntd.0011805 (PMC11556741; doi:10.1371/journal.pntd.0011805)
Supplement: S1 Table — (DOCX) [file pntd.0011805.s004.docx]

**Supporting Table 1.** Number of female tsetse with trypanosomes detected by microscopy or PCR in the midgut, salivary glands or mouthparts.

|  | |  | Midgut | | | Salivary glands | | | | Mouthparts | |
| --- | --- | --- | --- | --- | --- | --- | --- | --- | --- | --- | --- |
| OC | n | | | Micro | PCR | | Micro | PCR | Micro | | PCR |
| 0 | 201 | | | 2 | 12 | | 0 | 2 | 0 | | 4 |
| 1 | 296 | | | 4 | 10 | | 0 | 2 | 3 | | 5 |
| 2 | 212 | | | 3 | 5 | | 0 | 2 | 2 | | 3 |
| 3 | 181 | | | 2 | 5 | | 0 | 3 | 1 | | 4 |
| 4 | 449 | | | 6 | 16 | | 1 | 7 | 8 | | 16 |
| 5 | 393 | | | 5 | 16 | | 1 | 5 | 8 | | 9 |
| 6 | 129 | | | 4 | 6 | | 1 | 5 | 6 | | 9 |
| 7 | 70 | | | 1 | 4 | | 0 | 2 | 2 | | 5 |
