# Supplementary material for: Insights into trypanosomiasis transmission: Age, infection rates, and bloodmeal analysis of Glossina fuscipes fuscipes in N.W. Uganda
Source: PLoS Negl Trop Dis. 2024 Oct 31;18(10):e0011805. doi: 10.1371/journal.pntd.0011805 (PMC11556741; doi:10.1371/journal.pntd.0011805)
Supplement: S2 Table — (DOCX) [file pntd.0011805.s005.docx]

**Supporting Table 2.** Accession numbers for host bloodmeals detected, genotypes for each host varied across different hosts: cattle (*n*= 3), human (*n*= 7), forest cobra (*n*= 2), Nile monitor (*n*= 1), pig (*n*= 1), goat (*n*= 1) and sheep (*n*= 1).

| **Host** | **Accession numbers** |
| --- | --- |
| Cattle | PP445061 |
|  | PP445062 |
|  | PP445063 |
| Human | PP445064 |
|  | PP445065 |
|  | PP445066 |
|  | PP445067 |
|  | PP445068 |
|  | PP445069 |
|  | PP445070 |
| Forest cobra | PP445071 |
|  | PP445072 |
| Nile monitor | PP445073 |
| Pig | PP445074 |
| Goat | PP445075 |
| Sheep | PP445076 |
